# (*E*)-4-Bromo-*N*-(2-chloro­benzyl­idene)­aniline

**DOI:** 10.1107/S1600536811029977

**Published:** 2011-07-30

**Authors:** Chuang Wang

**Affiliations:** a State Key Laboratory of Pharmaceutical Biotechnology, School of Life Sciences, Nanjing University, Nanjing 210093, People’s Republic of China

## Abstract

In the title Schiff base mol­ecule, C_13_H_9_BrClN, the dihedral angle between the benzene rings is 49.8 (2)° and the mol­ecule has an *E* configuration about the C=N bond. In the crystal, there are no directional interactions but only van der Waals inter­molecular inter­action forces between neighbouring mol­ecules.

## Related literature

For the anti­bacterial activities of Schiff base compounds, see: El Masry *et al.* (2000 ▶). For the anti­cancer properties of Schiff base compounds, see: Dao *et al.* (2000 ▶). For related crystal structures, see: Sun *et al.* (2011*a* ▶,*b* ▶); Guo *et al.* (2011 ▶). For standard bond-length values, see: Allen *et al.* (1987 ▶).
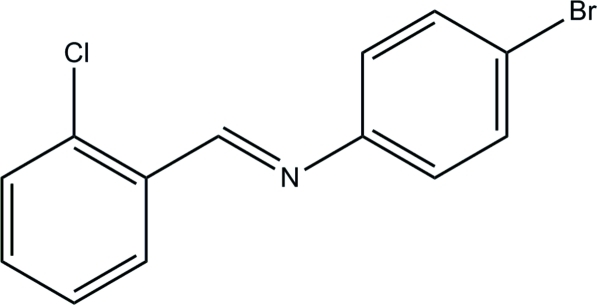


## Experimental

### 

#### Crystal data

C_13_H_9_BrClN
*M* *_r_* = 294.57
Monoclinic, 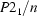

*a* = 15.243 (13) Å
*b* = 4.020 (4) Å
*c* = 20.142 (18) Å
β = 103.248 (8)°
*V* = 1201.4 (18) Å^3^
*Z* = 4
Mo *K*α radiation
μ = 3.61 mm^−1^
*T* = 296 K
0.25 × 0.23 × 0.21 mm

#### Data collection

Bruker APEXII CCD diffractometer
Absorption correction: multi-scan (*SADABS*; Sheldrick, 1996 ▶) *T*_min_ = 0.465, *T*_max_ = 0.518
7879 measured reflections
2219 independent reflections
1413 reflections with *I* > 2σ(*I*)
*R*_int_ = 0.049

#### Refinement

*R*[*F* ^2^ > 2σ(*F* ^2^)] = 0.048
*wR*(*F* ^2^) = 0.138
*S* = 1.04
2219 reflections
145 parameters
H-atom parameters constrained
Δρ_max_ = 0.54 e Å^−3^
Δρ_min_ = −0.43 e Å^−3^

### 

Data collection: *APEX2* (Bruker, 2007 ▶); cell refinement: *SAINT* (Bruker, 2007 ▶); data reduction: *SAINT*; program(s) used to solve structure: *SHELXS97* (Sheldrick, 2008 ▶); program(s) used to refine structure: *SHELXL97* (Sheldrick, 2008 ▶); molecular graphics: *SHELXTL* (Sheldrick, 2008 ▶); software used to prepare material for publication: *SHELXTL*.

## Supplementary Material

Crystal structure: contains datablock(s) global, I. DOI: 10.1107/S1600536811029977/su2298sup1.cif

Structure factors: contains datablock(s) I. DOI: 10.1107/S1600536811029977/su2298Isup2.hkl

Supplementary material file. DOI: 10.1107/S1600536811029977/su2298Isup3.cml

Additional supplementary materials:  crystallographic information; 3D view; checkCIF report

## Figures and Tables

**Fig. 1.**
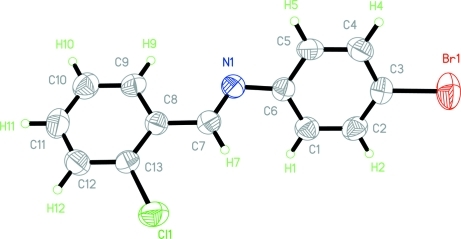
The molecular structure of the title molecule, with atom labels and displacement ellipsoids drawn at the 50% probability level.

## supplementary crystallographic information

### Comment

Schiff bases compounds have attracted a lot of attention for a long time, because of their applications as antibacterial (El Masry *et al.*, 2000), and anticancer (Dao *et al.*, 2000) agents. We report herein, on the crystal structure of the title new Schiff base compound.

The molecular structure of the title molecule is illustrated in Fig. 1. The geometric parameters agree well with those reported for similar structures (Sun *et al.*, 2011*a*,*b*; Guo *et al.*, 2011), and all the bond lengths are within normal ranges (Allen *et al.*, 1987). The dihedral angle between the two aromatic rings in the Schiff base molecule is 49.8 (2)°.

In the crystal, there are only van der Waals intermolecular forces between neighbouring molecules.

### Experimental

A mixture of 2-chlorobenzaldehyde (10 mmol), 4-bromoaniline (10 mmol) and methanol (50 ml) was refluxed for 6 h. It was then allowed to cool and was filtered. Recrystallization of the crude product from methanol yielded colourless crystals, suitable for X-ray diffraction analysis.

### Refinement

The H atoms were positioned geometrically and refined using the riding-model approximation: C—H = 0.93 Å, *U*_iso_(H) = 1.2*U*_eq_(C).

### Crystal data

**Table:**

C_13_H_9_BrClN	*F*(000) = 584
*M**_r_* = 294.57	*D*_x_ = 1.629 Mg m^−^^3^
Monoclinic, *P*2_1_/*n*	Mo *K*α radiation, λ = 0.71073 Å
Hall symbol: -P 2yn	Cell parameters from 1725 reflections
*a* = 15.243 (13) Å	θ = 2.8–21.7°
*b* = 4.020 (4) Å	µ = 3.61 mm^−^^1^
*c* = 20.142 (18) Å	*T* = 296 K
β = 103.248 (8)°	Block, colourless
*V* = 1201.4 (18) Å^3^	0.25 × 0.23 × 0.21 mm
*Z* = 4	

### Data collection

**Table:**

Bruker APEXII CCD diffractometer	2219 independent reflections
Radiation source: fine-focus sealed tube	1413 reflections with *I* > 2σ(*I*)
graphite	*R*_int_ = 0.049
φ and ω scans	θ_max_ = 25.5°, θ_min_ = 2.8°
Absorption correction: multi-scan (*SADABS*; Sheldrick, 1996)	*h* = −18→17
*T*_min_ = 0.465, *T*_max_ = 0.518	*k* = −4→4
7879 measured reflections	*l* = −24→24

### Refinement

**Table:**

Refinement on *F*^2^	Primary atom site location: structure-invariant direct methods
Least-squares matrix: full	Secondary atom site location: difference Fourier map
*R*[*F*^2^ > 2σ(*F*^2^)] = 0.048	Hydrogen site location: inferred from neighbouring sites
*wR*(*F*^2^) = 0.138	H-atom parameters constrained
*S* = 1.04	*w* = 1/[σ^2^(*F*_o_^2^) + (0.0738*P*)^2^ + 0.0187*P*] where *P* = (*F*_o_^2^ + 2*F*_c_^2^)/3
2219 reflections	(Δ/σ)_max_ = 0.001
145 parameters	Δρ_max_ = 0.54 e Å^−^^3^
0 restraints	Δρ_min_ = −0.43 e Å^−^^3^

### Special details

**Table:**

Geometry. All e.s.d.'s (except the e.s.d. in the dihedral angle between two l.s. planes) are estimated using the full covariance matrix. The cell e.s.d.'s are taken into account individually in the estimation of e.s.d.'s in distances, angles and torsion angles; correlations between e.s.d.'s in cell parameters are only used when they are defined by crystal symmetry. An approximate (isotropic) treatment of cell e.s.d.'s is used for estimating e.s.d.'s involving l.s. planes.
Refinement. Refinement of *F*^2^ against ALL reflections. The weighted *R*-factor *wR* and goodness of fit *S* are based on *F*^2^, conventional *R*-factors *R* are based on *F*, with *F* set to zero for negative *F*^2^. The threshold expression of *F*^2^ > σ(*F*^2^) is used only for calculating *R*-factors(gt) *etc*. and is not relevant to the choice of reflections for refinement. *R*-factors based on *F*^2^ are statistically about twice as large as those based on *F*, and *R*- factors based on ALL data will be even larger.

### Fractional atomic coordinates and isotropic or equivalent isotropic displacement parameters (Å^2^)

**Table:**

	*x*	*y*	*z*	*U*_iso_*/*U*_eq_	
Br1	0.28006 (4)	0.71850 (15)	1.03703 (3)	0.0824 (3)	
C1	0.4330 (3)	0.6328 (11)	0.8929 (2)	0.0499 (11)	
H1	0.4917	0.6854	0.8906	0.060*	
C2	0.4042 (3)	0.6997 (11)	0.9513 (2)	0.0544 (12)	
H2	0.4431	0.8014	0.9880	0.065*	
C3	0.3186 (3)	0.6179 (11)	0.9560 (2)	0.0501 (11)	
C4	0.2584 (3)	0.4713 (12)	0.9009 (2)	0.0544 (12)	
H4	0.2003	0.4154	0.9041	0.065*	
C5	0.2870 (3)	0.4110 (12)	0.8416 (2)	0.0527 (12)	
H5	0.2469	0.3189	0.8042	0.063*	
C6	0.3744 (3)	0.4854 (11)	0.8366 (2)	0.0440 (10)	
C7	0.4763 (3)	0.2977 (10)	0.7752 (2)	0.0479 (11)	
H7	0.5136	0.2425	0.8171	0.057*	
C8	0.5086 (3)	0.2397 (10)	0.7133 (2)	0.0438 (10)	
C9	0.4571 (3)	0.3496 (11)	0.6495 (2)	0.0506 (11)	
H9	0.4020	0.4532	0.6473	0.061*	
C10	0.4871 (4)	0.3061 (12)	0.5909 (3)	0.0603 (13)	
H10	0.4520	0.3800	0.5495	0.072*	
C11	0.5684 (4)	0.1546 (13)	0.5928 (3)	0.0633 (14)	
H11	0.5886	0.1297	0.5528	0.076*	
C12	0.6206 (3)	0.0384 (12)	0.6542 (2)	0.0575 (13)	
H12	0.6753	−0.0670	0.6557	0.069*	
C13	0.5897 (3)	0.0821 (11)	0.7133 (2)	0.0452 (10)	
Cl1	0.65673 (8)	−0.0722 (3)	0.78942 (6)	0.0647 (4)	
N1	0.3988 (2)	0.4211 (9)	0.77412 (18)	0.0491 (9)	

### Atomic displacement parameters (Å^2^)

**Table:**

	*U*^11^	*U*^22^	*U*^33^	*U*^12^	*U*^13^	*U*^23^
Br1	0.1072 (6)	0.0843 (5)	0.0678 (4)	−0.0119 (3)	0.0453 (4)	−0.0171 (3)
C1	0.046 (2)	0.052 (3)	0.052 (3)	−0.006 (2)	0.011 (2)	0.002 (2)
C2	0.054 (3)	0.057 (3)	0.049 (3)	−0.011 (2)	0.004 (2)	−0.005 (2)
C3	0.061 (3)	0.046 (3)	0.045 (3)	0.002 (2)	0.016 (2)	0.000 (2)
C4	0.043 (2)	0.057 (3)	0.065 (3)	−0.004 (2)	0.015 (2)	−0.010 (2)
C5	0.038 (3)	0.062 (3)	0.053 (3)	0.000 (2)	0.001 (2)	−0.004 (2)
C6	0.048 (3)	0.042 (3)	0.042 (2)	0.006 (2)	0.009 (2)	0.002 (2)
C7	0.047 (3)	0.051 (3)	0.045 (3)	0.000 (2)	0.008 (2)	0.000 (2)
C8	0.044 (2)	0.042 (3)	0.045 (3)	0.000 (2)	0.010 (2)	−0.002 (2)
C9	0.049 (3)	0.054 (3)	0.045 (3)	0.003 (2)	0.003 (2)	0.005 (2)
C10	0.064 (3)	0.065 (3)	0.050 (3)	−0.001 (3)	0.010 (2)	0.004 (2)
C11	0.073 (3)	0.068 (3)	0.053 (3)	−0.006 (3)	0.022 (3)	−0.012 (3)
C12	0.046 (3)	0.062 (3)	0.066 (3)	−0.001 (2)	0.015 (2)	−0.014 (3)
C13	0.043 (2)	0.047 (3)	0.043 (2)	−0.005 (2)	0.005 (2)	−0.005 (2)
Cl1	0.0567 (7)	0.0718 (9)	0.0585 (8)	0.0150 (6)	−0.0019 (6)	−0.0042 (6)
N1	0.046 (2)	0.052 (2)	0.048 (2)	0.0069 (19)	0.0096 (17)	−0.0027 (18)

### Geometric parameters (Å, °)

**Table:**

Br1—C3	1.900 (5)	C7—C8	1.461 (6)
C1—C2	1.374 (6)	C7—H7	0.9300
C1—C6	1.404 (6)	C8—C13	1.390 (6)
C1—H1	0.9300	C8—C9	1.414 (6)
C2—C3	1.369 (7)	C9—C10	1.371 (7)
C2—H2	0.9300	C9—H9	0.9300
C3—C4	1.399 (6)	C10—C11	1.374 (8)
C4—C5	1.384 (6)	C10—H10	0.9300
C4—H4	0.9300	C11—C12	1.390 (7)
C5—C6	1.391 (6)	C11—H11	0.9300
C5—H5	0.9300	C12—C13	1.387 (6)
C6—N1	1.416 (5)	C12—H12	0.9300
C7—N1	1.276 (5)	C13—Cl1	1.750 (4)
			
C2—C1—C6	120.3 (4)	C8—C7—H7	118.7
C2—C1—H1	119.9	C13—C8—C9	116.8 (4)
C6—C1—H1	119.9	C13—C8—C7	123.2 (4)
C3—C2—C1	120.6 (4)	C9—C8—C7	120.0 (4)
C3—C2—H2	119.7	C10—C9—C8	121.2 (4)
C1—C2—H2	119.7	C10—C9—H9	119.4
C2—C3—C4	120.6 (4)	C8—C9—H9	119.4
C2—C3—Br1	119.7 (4)	C9—C10—C11	120.7 (5)
C4—C3—Br1	119.7 (3)	C9—C10—H10	119.7
C5—C4—C3	118.7 (4)	C11—C10—H10	119.7
C5—C4—H4	120.7	C10—C11—C12	120.1 (5)
C3—C4—H4	120.7	C10—C11—H11	119.9
C4—C5—C6	121.4 (4)	C12—C11—H11	119.9
C4—C5—H5	119.3	C13—C12—C11	118.9 (4)
C6—C5—H5	119.3	C13—C12—H12	120.5
C5—C6—C1	118.4 (4)	C11—C12—H12	120.5
C5—C6—N1	118.4 (4)	C12—C13—C8	122.4 (4)
C1—C6—N1	123.1 (4)	C12—C13—Cl1	117.5 (3)
N1—C7—C8	122.6 (4)	C8—C13—Cl1	120.1 (3)
N1—C7—H7	118.7	C7—N1—C6	119.1 (4)
			
C6—C1—C2—C3	1.3 (7)	C7—C8—C9—C10	178.2 (4)
C1—C2—C3—C4	−1.4 (7)	C8—C9—C10—C11	−0.2 (7)
C1—C2—C3—Br1	−179.2 (3)	C9—C10—C11—C12	1.1 (8)
C2—C3—C4—C5	−0.1 (7)	C10—C11—C12—C13	−0.8 (7)
Br1—C3—C4—C5	177.7 (4)	C11—C12—C13—C8	−0.3 (7)
C3—C4—C5—C6	1.6 (7)	C11—C12—C13—Cl1	179.2 (4)
C4—C5—C6—C1	−1.7 (7)	C9—C8—C13—C12	1.2 (6)
C4—C5—C6—N1	−179.3 (4)	C7—C8—C13—C12	−178.0 (4)
C2—C1—C6—C5	0.2 (6)	C9—C8—C13—Cl1	−178.4 (3)
C2—C1—C6—N1	177.7 (4)	C7—C8—C13—Cl1	2.5 (6)
N1—C7—C8—C13	−174.9 (4)	C8—C7—N1—C6	−176.8 (4)
N1—C7—C8—C9	6.0 (6)	C5—C6—N1—C7	−139.1 (4)
C13—C8—C9—C10	−1.0 (7)	C1—C6—N1—C7	43.5 (6)
